# The beneficial effects of alpha‐tocopherol on intestinal function and the expression of tight junction proteins in differentiated segments of the intestine in piglets

**DOI:** 10.1002/fsn3.3103

**Published:** 2022-10-30

**Authors:** Yanjun Huang, Caimei He, Zheng Hu, Xuetong Chu, Sichun Zhou, Xin Hu, Jun Deng, Di Xiao, Ting Tao, Huansheng Yang, Alex F. Chen, Yulong Yin, Xiaoping Yang

**Affiliations:** ^1^ Key Laboratory for Study and Discovery of Small Targeted Molecules of Hunan Province Department of Pharmacy, School of Medicine Hunan Normal University Changsha China; ^2^ Research Center for Healthy Breeding of Livestock and Poultry Hunan Engineering and Research Center of Animal and Poultry Science and Key Laboratory for Agro‐ecological Processes in Subtropical Region Institute of Subtropical Agriculture, The Chinese Academy of Sciences Changsha China

**Keywords:** nutritional supplement, piglets, TEER, tight junction protein, α‐Tocopherol

## Abstract

Alpha (α)‐tocopherol is a major component of dietary vitamin E. Despite being one of the most widely used food supplements in both animals and humans, its role in intestinal functions remains unknown. We were able to examine and accurately demonstrate its permeability effect in vitro and its differentiated effect on tight junction expression in different segments of the intestine in vivo using cultured intestinal porcine epithelial cell line (IPEC‐J2) and piglets. A cultured IPEC‐J2 demonstrated that α‐tocopherol upregulated the expression of tight junction proteins and improved their integrity, with a maximum effect at concentrations ranging from 20 to 40 μmol/L. In vivo data from weaned pigs fed different doses of α‐tocopherol for 2 weeks revealed that α‐tocopherol effectively increases the expression of tight junction proteins in all sections of the intestinal mucosa, with the highest effect on the duodenum at an optimum dose of 20–50 mg/kg. In contrast, α‐tocopherol did not affect intestinal inflammation. These findings suggest that α‐tocopherol maintains intestinal integrity and increases the expression of tight junction proteins both in vitro and in vivo.

## Graphical abstract

we disclose that α‐tocopherol has profound effect on enhancing expression of tight junction proteins in the intestinal tract and inhibiting permeability in the intestine in normal healthy conditions, both in vivo and in vitro. The detailed mechanisms how α‐tocopherol exerts effect on the intestinal barrier and intestinal inflammation in diseased conditions deserve further study.

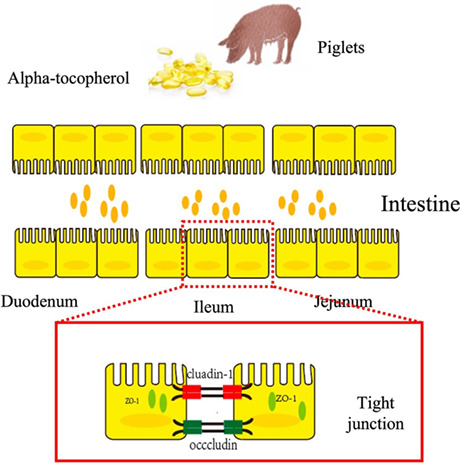

## 1 INTRODUCTION

α‐Tocopherol, one of the main components of vitamin E, is considered an essential dietary nutrient for humans and animals. Numerous evidence demonstrates the biological function and protective effects of α‐tocopherol consumption on human health, but the molecular mechanisms underlying its function of health benefits remain largely unknown (Burbank et al., 2018; Dolfi et al., 2013; Kim et al., 2016; Shibata et al., 2016). Although many studies focus on its anti‐oxidative effect (Alshiek et al., 2017), the preferential localization of α‐tocopherol in the cell membranes has attracted profound attention due to its recently recognized functional role as a membrane stabilizer (Ehsan et al., 2018; Zingg, 2019). Thus, α‐tocopherol may play an important role in maintaining physiological homeostasis in healthy conditions by influencing cellular integrity, which needs further investigation.

The intestinal barrier and intestinal permeability are well‐known biomarkers of cellular integrity that significantly affect health (Shaw et al., 2012). Tight junction forms the paracellular epithelial barrier to ions and solutes, separating tissue spaces and producing well‐defined transcellular absorption and secretion that maintain the intestinal permeability balance and protect intestinal barrier functions (Ivanov, 2012). Transmembrane proteins that physically form the sealing contacts, such as members of the Claudin and plasma‐membrane protein Occludin families, are critical barrier components (Brandner et al., 2015). Transmembrane proteins bind to scaffolding proteins, including zonula occludens 1 (Zo‐1), which interacts with cytoskeletal elements to regulate junctional integrity (Pummi et al., 2001). Thus, tight junction proteins like Claudin, Occludin, and Zo‐1 are essential components of intestinal epithelia and produce synergistic effects to maintain physiological homeostasis in mammals (Pearce et al., 2018). Recent evidence from rat and cellular models demonstrates that α‐tocopherol repairs hyperoxia‐ and hyperchromium‐induced tight junction protein damage (Simon et al., 1999; Soini, 2012; Zahraoui, 2004). However, it has not yet been determined whether α‐tocopherol affects intestinal tight junction proteins under normal physiological conditions.

Numerous studies have undoubtedly demonstrated that α‐tocopherol has anti‐inflammatory effects on cystic fibrosis, chronic inflammation, esophageal carcinogenesis, and obesity‐induced insulin resistance (McMorrow et al., 2018; Sagel et al., 2018; Yang et al., 2018). These studies were conducted in disease‐related conditions (Lee & Han, 2018; Liu et al., 2019). However, as a nutritional supplement, α‐tocopherol has been used in healthy conditions that have been rarely studied. Furthermore, low α‐tocopherol concentration could not achieve the desired results, while overdose would result in severe side effects (Ikegami et al., 2017; Vega‐Mata et al., 2017). Therefore, determining the optimal supplemental dose of α‐tocopherol is significantly important for clinically maintaining both human and animal health.

Duodenum is the proximal part of the small intestine as the continuation of the stomach. Food is discharged from the stomach into the duodenum because it is directly connected to the stomach (Fang et al., 2018). The following two segments of the small intestine are the jejunum and ileum (Tripathy et al., 2017). They each play a distinguished role in nutritional absorption. Our previous study found that α‐tocopherol inhibited the proliferation of intestinal epithelial cells (Chen et al., 2019). In the present study, we try to elucidate the effect of α‐tocopherol on intestinal tight junctions both in vitro and in vivo.

Furthermore, the mucosa in the intestine serves as the contact surface for nutrient absorption (Pu et al., 2020). Taking advantage of piglets, one of the most commonly used large animals, we were able to precisely examine and accurately demonstrate the differentiated effect of α‐tocopherol on tight junction expression in different segments of the intestine in vivo. To validate whether the α‐tocopherol supplement has an inflammatory impact on the intestine, changes in interleukin 6 (IL‐6) and tumor necrosis factor alpha (TNF‐α) levels were also measured.

## 2 MATERIALS AND METHODS

### 2.1 Reagents and cell culture

α‐Tocopherol (Cat: 258024) (Sigma‐Aldrich in China, Shanghai, China) was dissolved in dimethyl sulfoxide (DMSO) to prepare the stock solution of 100 mmol/L for cell culture experiments and added to the regular diet for animal studies. Antibodies against the following target proteins: Claudin 1, Occludin, Zo‐1, phospho‐nuclear factor kappa B (p‐NF‐κB), IL‐6, TNF‐α, and β‐actin were obtained from Cell Signaling Technology (Beverly, MA, USA). The intestinal porcine epithelial cell line (IPEC‐J2 cell line) from Animal Nutrition & Human Health Laboratory was cultured in Dulbecco's modified Eagle's medium (DMEM) (Hyclone, Logan, UT, USA) supplemented with 10% of fetal calf serum (Hyclone, Logan, UT, USA) and 1% of penicillin–streptomycin at 37°C, in humidified air containing 5% of CO_2_.

### 2.2 Animals and treatments

A total of 42 piglets [(Landrace × Yorkshire) × Duroc] with average initial body weight (BW) of 6.40 ± 0.44 kg were weaned at 21 days of age and randomly assigned to one of the five treatment groups based on sex and BW: (1) only basal diet (control), (2) basal diet +10 mg/kg α‐tocopherol, (3) basal diet +20 mg/kg α‐tocopherol, (4) basal diet +50 mg/kg α‐tocopherol, and (5) basal diet +100 mg/kg α‐tocopherol.

Table 1 shows the composition of the basal diet. For a 14‐day experimental period, each treatment had 14 replicates, with seven males and seven females. Diets based on corn and soybean meal had comparable nutrient levels but differed in their α‐tocopherol contents. For 2 weeks, animals were allowed to drink water and were fed six times per day. To avoid individual differences, the average feed intake and weight gain of each piglet were recorded daily. This animal protocol (No. ISA 2015–007) was reviewed and approved by the Institutional Animal Care and Use Committee.

**TABLE 1:** Composition of basal piglet diets

Item	Composition (%)
Corn	42.85
Extruded corn	20
Soybean meal	10
Soy protein concentrate	2.88
Whey powder	10
Fish meal	3
Spray‐dried porcine plasma	5
L‐Lys	0.55
DL‐Met	0.12
L‐Thr	0.13
L‐Try	0.04
Soybean oil	2.55
Limestone	1.08
Dicalcium phosphate	0.7
Choline chloride	0.1
Antioxidants	0.05
Citric acid	0.3
Mineral premixaa	0.15
Vitamin premixab	0.5
Total	100
Calculated composition CP, %	18
ME, kcal/kg	3400
Ca, %	0.8
Available *p*, %	0.36
Lys, c %	1.35
Met, c %	0.39
SAA, c, d %	0.74
Thr, c %	0.79
Trp, c %	0.22

### 2.3 Quantitative real‐time (RT)‐PCR

In a final volume of 20 μl, the polymerase chain reaction (PCR) system contained 5.0 μl of SYBR Green qPCR Mix, 0.2 μl of complementary DNA (cDNA), 0.3 μl of each primer, and 4.2 μl of double‐distilled water. Table 2 contains detailed information about each primer. Each sample was tested in triplicate, and the internal standard for PCR was the housekeeping gene glyceraldehyde 3‐phosphate dehydrogenase (GAPDH). The same pig GAPDH primer was used in quantitative real‐time PCR with a RealMasterMix SYBP ROX (5 Prime) according to the manufacturer's protocols. Primer sequences (Sangon Biotech Co., Ltd.) are listed in Table 2.

**TABLE 2:** List of primers used in reverse transcription polymerase chain reaction (RT‐PCR) both in vivo and in vitro

Gene	Forward primer (5′–3′)	Reverse primer (3′–5′)
Claudin 1	CTGGAAATCCTCGGCCTCGT	GCCGTCACGATGTTGTGGTC
Zo‐1	GGCCCTTACCTTTCGCCTGA	GCCTCAGGGCTTGGTGTTCT
Occludin	TTGCCTGGGACGAGGCTATG	ATCCCTTTGCTGCTCGTGGA
TNF‐α	ATTCAGGGATGTGTGGCCTG	CCAGATGTCCCAGGTTGCAT
IL‐6	TGGATAAGCTGCAGTCACAG	ATTATCCGAATGGCCCTCAG
GAPDH	GAAGGTCGGAGTGAACGGAT	CTGGCATTGACTGGGGTCAT
β‐Actin	CTTCTTGGGCATGGAGTC	TAGAGGTCCTTCCTGATGT

### 2.4 Protein characterization

Western blot analysis was performed using the previously described standard procedure. The primary antibody was added to BSA (bovine serum albumin) and incubated overnight at 4°C. Then it was washed with Tris‐buffered saline (TBS)/0.05% Tween 20 before adding the secondary antibody and incubated for an additional 1 h at room temperature. The membrane was washed three times more before adding Pierce Super Signal chemiluminescent substrate (Rockford, IL, USA) and imaged immediately on ChemiDoc (Bio‐Rad, Hercules, CA, USA). The images were scanned with an Epson Perfection V500 Photo scanner and quantified by ImageJ (NIH, Bethesda, MD, USA).

### 2.5 Immunofluorescence

The IPEC‐J2 cell line was cultured on glass coverslips. After harvesting, cells were fixed with 4% paraformaldehyde for 15 min at room temperature and permeabilized with 0.1% Triton for 10 min. The cells were blocked with 5% bovine serum and incubated overnight at 4°C with the indicated primary antibody. On the second day, coverslips were washed three times with phosphate‐buffered saline (PBS), incubated with the appropriate secondary antibody for 45 min at room temperature, and stained with 4,6‐diamidino‐2‐phenylindole (DAPI). Light microscopy was used to capture immunofluorescent images (Leica, DM3000, Germany).

### 2.6 Cell permeability: Measurement of transepithelial electrical resistance (TEER assay)

The IPEC‐J2 cells were seeded (1 × 105 cells per cm^2^) in a transwell chamber with 4.5 μm pores (Costar, Coring Inc, New York, NY, USA) that had been placed in a six‐well plate. The other transwell chamber remained empty. After confluence, cells were differentiated and polarized for 7–10 days in the culture medium. Before and after all treatments, TEER was used to assess cell monolayer integrity. It was measured with a chopstick electrode (Millicell ERS‐2, EMD Millipore Corporation, Billerica, MA) on an epithelial volt‐ohm meter. The electrode was immersed at a 90° angle with one tip in the basolateral chamber and the other in the apical chamber. Care was taken to avoid electrode contact with the monolayer, and measurements were taken in triplicate for each monolayer. An insert without cells was used as a blank, and its mean resistance was subtracted from all samples. Unit area resistance was then calculated by dividing resistance values by the effective membrane area (4.5 cm^2^).

### 2.7 Extraction of RNA and real‐time quantitative PCR of intestinal mucosa and inner muscle tissue samples

Total RNA was extracted from intestinal mucosa and inner muscle tissues of the ileum, jejunum, and duodenum using Trizol (Sangon Biotech, Shanghai, China) and then treated with deoxyribonucelase I (DNase) I to remove traces of DNA.

A previously described detailed procedure of RT‐PCR was followed. Primers for TNF‐α, IL‐6, Claudin 1, Zo‐1, Occludin, and β‐actin were designed with Primer 5.0.

### 2.8 Enzyme‐linked immunosorbent assays (ELISAs)

Allow serum samples to clot for 2 h at room temperature or overnight at 4°C before centrifugation at approximately 1000 × *g* for 20 min. Assay freshly prepared serum immediately or store samples in an aliquot at −80°C for later use. Avoid repeated freeze/thaw cycles. The α‐tocopherol ELISA kit was purchased from Shanghai Yu Yu Biotechnology Co. Ltd. The assay was performed according to the Kit Instruction Manual. In the end, a stop solution was added, and the color changed from blue to yellow. A microplate reader (BioTek Synergy HTX, Vermont, USA) measured the intensity at 450 nm. The calibration standards were assayed concurrently with the samples, allowing the operator to produce a standard curve of optimum density (OD) versus α‐tocopherol concentration. The α‐tocopherol concentrations in the samples were then determined by comparing the OD of samples to the standard curve.

### 2.9 Statistical analysis

All data are presented as mean ± SD. Analysis of variance (anova) was used for statistical analyses, and statistical significance was assumed at *p* < .05.

## 3 RESULTS

### 3.1 α‐Tocopherol increased the expression of tight junction proteins in IPEC‐J2 cell line detected by RT‐PCR and Western blot

**FIGURE 1:**
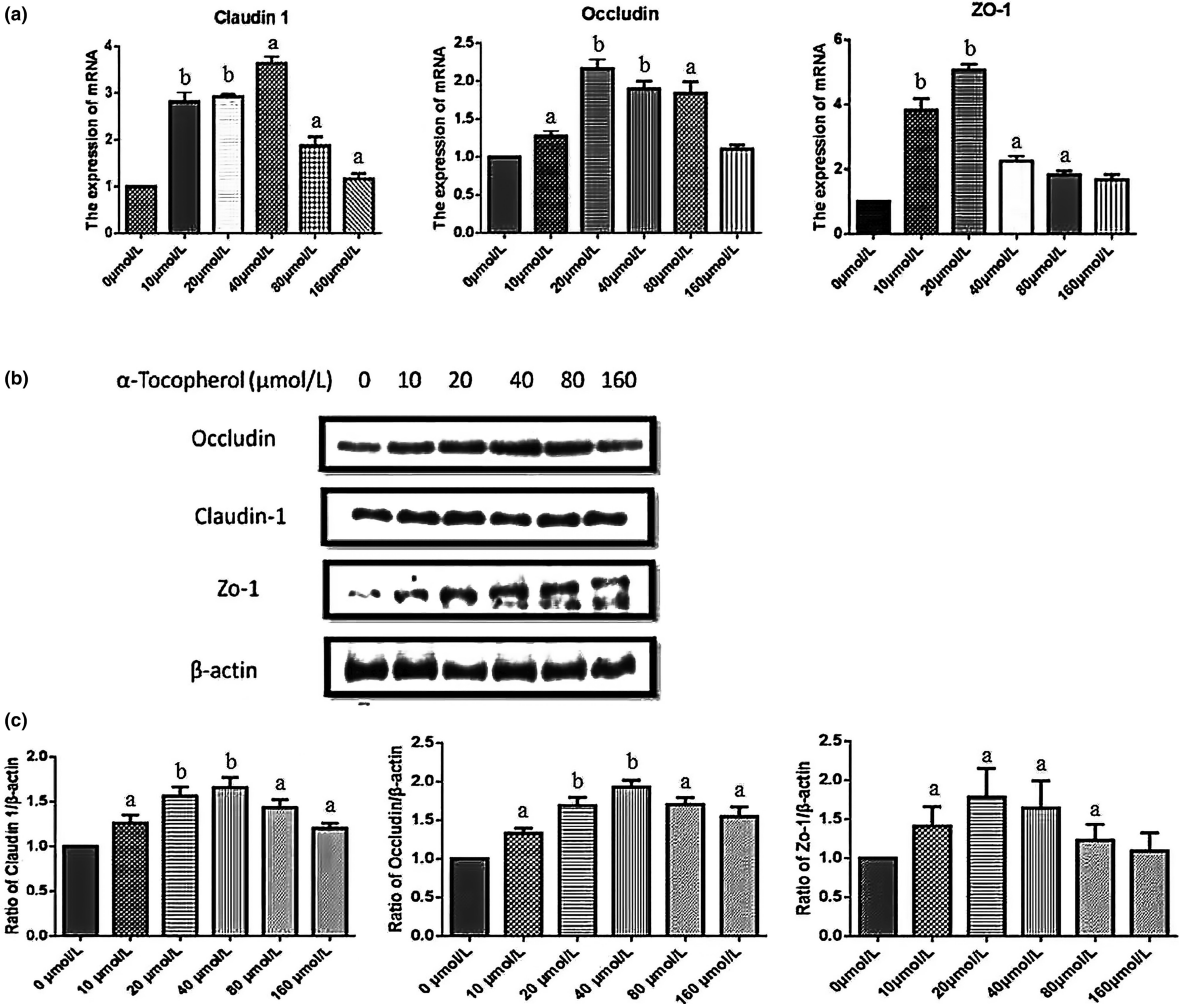
α‐Tocopherol increases the expression of tight junction proteins. (a) Reverse transcription‐polymerase chain reaction (RT‐PCR) results of Claudin 1, zonula occludens 1 (Zo‐1), and Occludin of intestinal porcine epithelial cell line (IPEC‐J2). (b) Western blot results of Claudin 1, Zo‐1, and Occludin of intestinal epithelial cell line IPEC‐J2. β‐Actin was included as a loading control. (c) The ratio of three tight junction proteins to β‐actin was calculated by the band density of Western blots of each cell line using imagej software. (a < 0.05, b < 0.01 vs. control). The data were represented as means ± SD.

The IPEC‐J2 cell line was treated with α‐tocopherol at doses of 0–160 μmol/L for 24 h. Upregulation of Occludin, Claudin 1, and Zo‐1 expression was measured through RT‐PCR. The most effective concentrations of α‐tocopherol are 20–40 μmol/L (Figure 1a). Protein levels of Occludin, Claudin 1, and Zo‐1 measured by western blotting revealed the same patterns (Figure 1b). These findings indicated that α‐tocopherol increases the tight junction proteins such as Occludin, Claudin 1, and Zo‐1.

### 3.2 α‐Tocopherol increased the protein expression of tight junction and integrity of tight junction detected by immunofluorescence images

**FIGURE 2:**
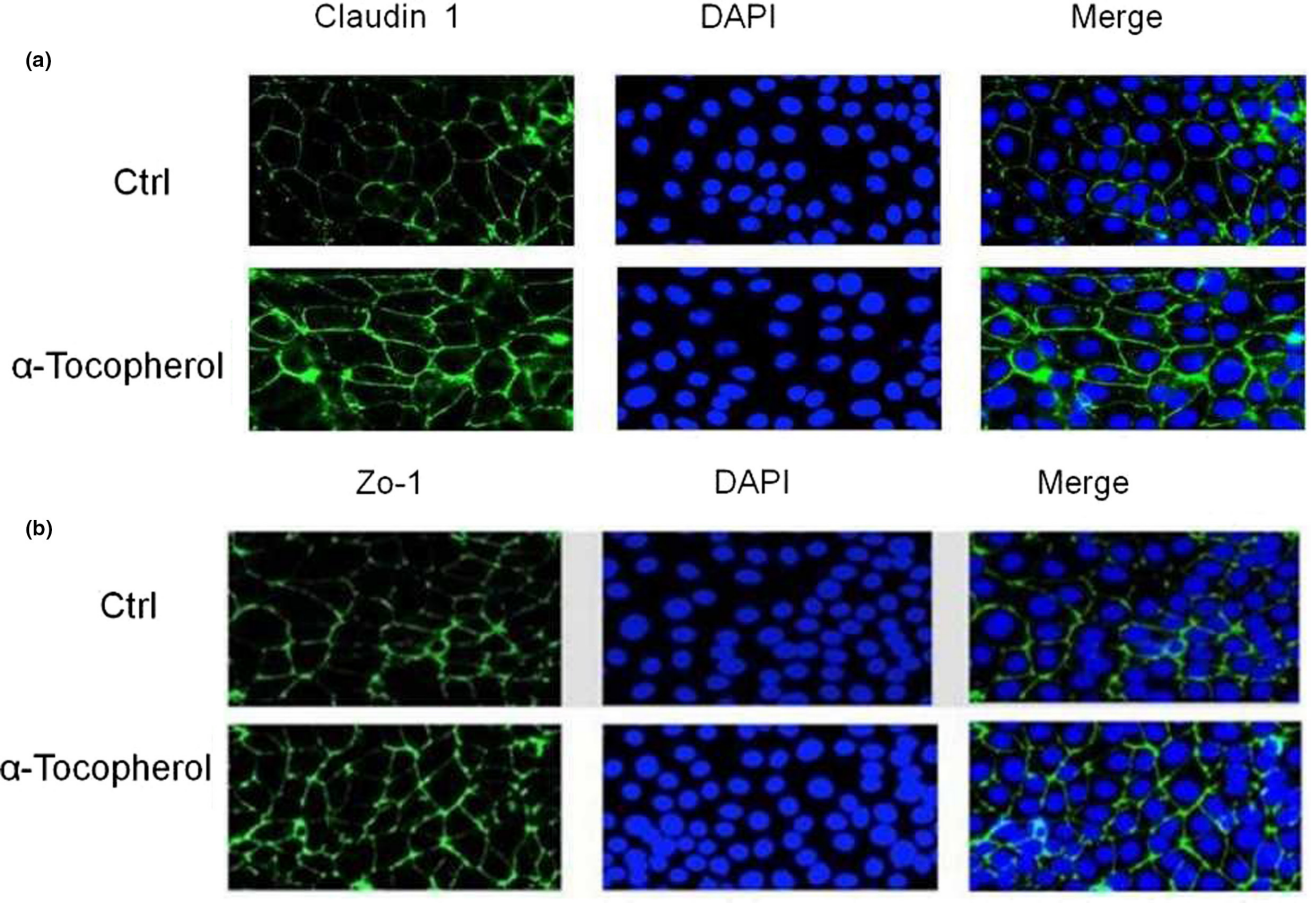
The expression and localization of tight junction proteins were detected by immunofluorescence. Immunofluorescence images were collected to compare the expression of Claudin 1 (a) and zonula occludens 1 (Zo‐1) (b) of control group with those of Vitamin E (VE)‐treated group (magnification 400X).

Treatment of IPEC‐J2 cell line with α‐tocopherol significantly increased the fluorescence intensity of Claudin 1 and Zo‐1 proteins, consistent with the results of RT‐PCR and western blot described above (Figure 2). Meanwhile, immunofluorescent images reveal that the integrity of the tight junction proteins Claudin 1 and Zo‐1, distinguished by cellular structure patterns, was significantly improved (Figure 2). The maximum effect on these proteins was observed at 20 μmol/L α‐tocopherol, suggesting an optimal concentration at the cellular level.

### 3.3 α‐Tocopherol had no effect on the expression of phospho‐NF‐κB

**FIGURE 3:**
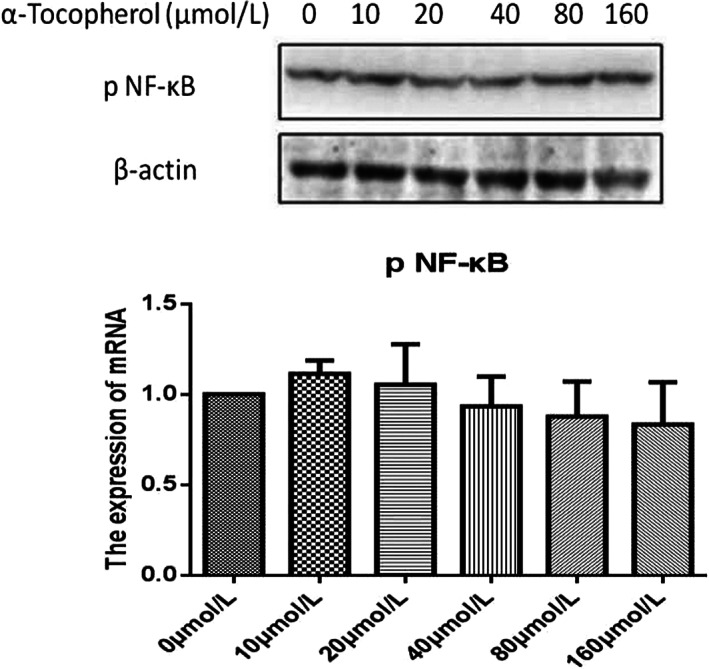
α‐Tocopherol does NOT exert any anti‐inflammatory effect on cytokines. Lysates of the intestinal porcine epithelial cell line (IPEC‐J2) cells treated with α‐tocopherol at various concentrations (0–160 μmol/L) were Western‐blotted to assess the levels of phosphorylated proteins. (a) Represents Western blotting of phospho‐nuclear factor kappa B (phospho‐NF‐κB) of the IPEC‐J2 cell line. β‐Actin was included as a loading control. (b) The ratio of three tight junction proteins to β‐actin was calculated by the band density of Western blots of each cell line using ImageJ software. The data were represented as means ± SD.

One of the well‐known anti‐inflammatory cytokines involved in the pathogenesis of the intestinal disease is phospho‐NF‐κB. In the present study, we found that treating IPEC‐J2 cells with α‐tocopherol at doses ranging from 0 to 160 μmol/L for 24 h had no significant effect on the protein expression of phospho‐NF‐κB (Figure 3a). These findings illustrate that α‐tocopherol does not affect the expression of phospho‐NF‐κB under normal cell conditions, in contrast to diseased cell conditions.

### 3.4 α‐Tocopherol increased transepithelial electrical resistance (TEER)

**FIGURE 4:**
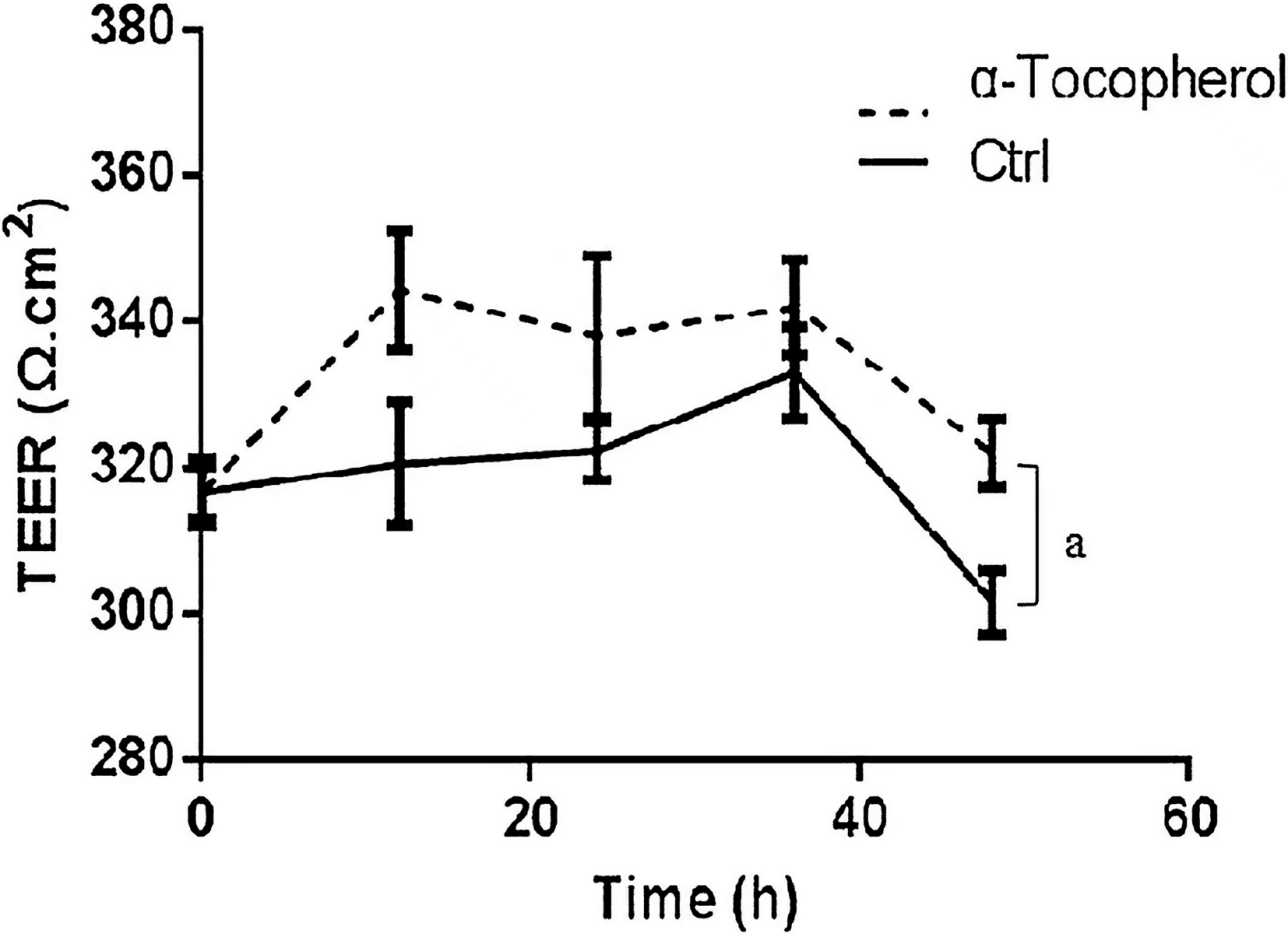
α‐Tocopherol treatment increases transepithelial electrical resistance (TEER). TEER measurements under basal conditions show stably increased TEER values after α‐tocopherol treatment. The data were represented as mean TEER value ± SD (*n* = 3, a < 0.05 vs. control).

TEER values increased slightly but significantly in the 20 μmol/L α‐tocopherol‐treated groups compared to the control group (Figure 4), indicating that α‐tocopherol increases TEER in the IPEC‐J2 cell line while reducing cell permeability and protecting tight junction integrity.

### 3.5 α‐Tocopherol levels were detectable in serum after 100 mg/kg α‐tocopherol administration in piglets

**FIGURE 5:**
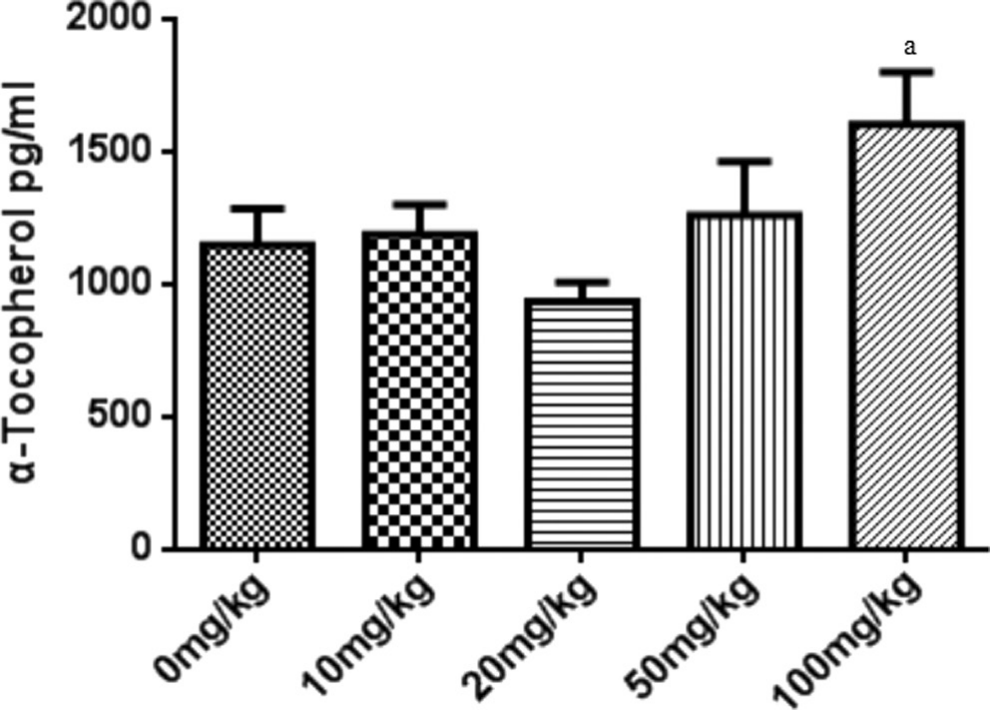
Porcine α‐tocopherol concentrations in serum do not increase until the dose is higher than 100 mg/kg. The relative concentrations of α‐tocopherol in the samples from weaned pig's serum were detected in microplate reader (BioTek, SYNERGY HTX, VT, USA) at 490 nm by the α‐tocopherol enzyme‐linked immunosorbent assay (ELISA) kit. All data were represented as mean ± SD (*n* = 3, a < 0.05 vs. control).

The serum from different groups of piglets was collected, and the levels of α‐tocopherol were measured by elisa. It was found that the serum α–tocopherol concentrations in the 10, 20, and 50 mg/kg groups were not significantly different from the control group (0 mg/kg group). However, α‐tocopherol concentration in serum was significantly higher in the 100 mg/kg treated group than in the control group. These findings indicate that the minimum dose for a detectable increase of α‐tocopherol in piglet serum is 100 mg/kg (Figure 5).

### 3.6 The effects of α‐tocopherol on intestinal inflammatory markers in weaned pig

**FIGURE 6:**
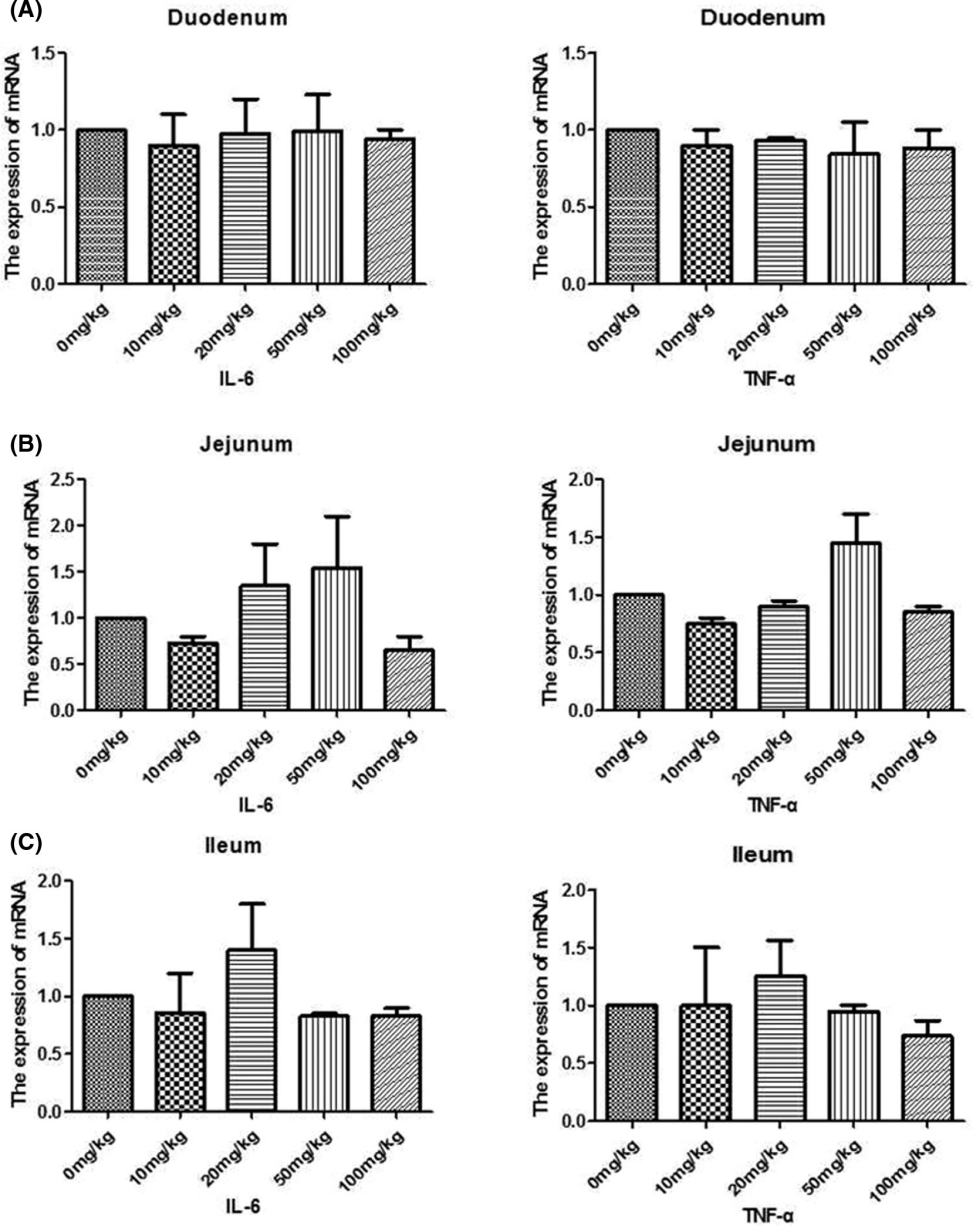
Effects of α‐tocopherol on inflammatory factors such as interleukin 6 (IL‐6) and tumor necrosis factor alpha (TNF‐α) in ileum, jejunum, and duodenum. Detection of intestinal inflammation‐related gene expression in weaned piglets by reverse transcription polymerase chain reaction (RT‐PCR) on the intestinal mucosa in duodenum (a), jejunum (b), and ileum (c).

To investigate the effect of α‐tocopherol on inflammatory biomarkers, the gene expression of inflammatory cytokines IL‐6 and TNF‐α was determined in different intestinal mucosa groups through RT‐PCR. It was found that α‐tocopherol had no statistically significant effect on inflammatory factors in the intestinal mucosa of piglets fed (Figure 6). This finding is consistent with the previous results at in vitro cellular levels.

### 3.7 The effects of α‐tocopherol on intestinal mucosal tight junction proteins

**FIGURE 7:**
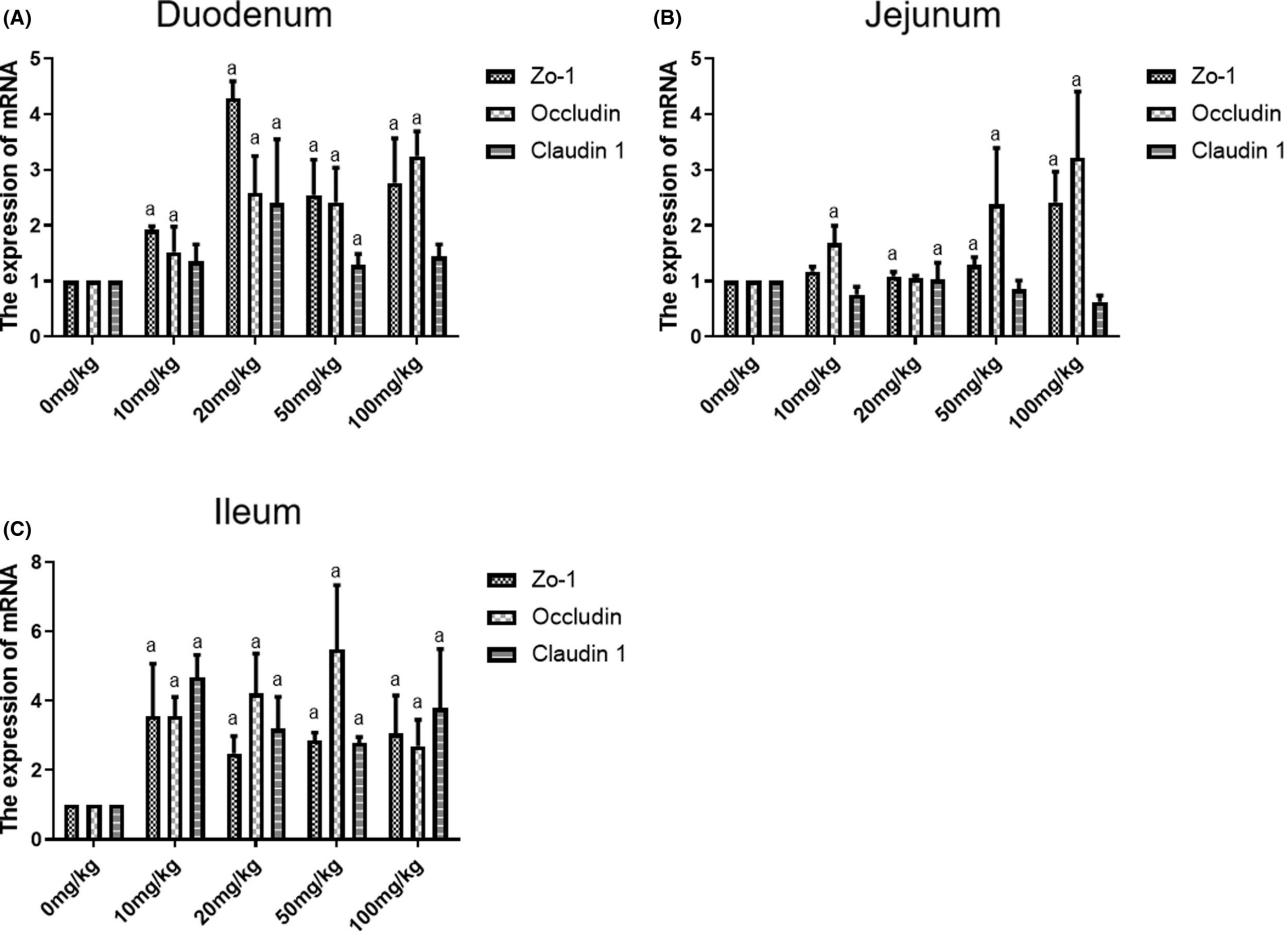
Effects of different concentrations of α‐tocopherol on intestinal mucosal tight junction proteins. Detection of intestinal tight junction proteins such as Claudin 1, zonula occludens (Zo‐1), and Occludin expression in weaned piglets by reverse transcription polymerase chain reaction (RT‐PCR) on the intestinal mucosa in duodenum (a), jejunum (b), and ileum (c). All data were represented as mean ± SD (*n* = 3, a < 0.05 vs. control).

Occludin and Zo‐1 in mucosa from all segments, including duodenum, ileum, and jejunum, were upregulated by α‐tocopherol in the range of 0–100 mg/kg, as shown in Figure 7. Claudin 1 expression was upregulated in the mucosa of the duodenum and ileum but not in the jejunum. The optimal dose range for the upregulatory effect of α‐tocopherol on tight junction proteins at mucosa is 20–50 mg/kg. In the duodenum and ileum, the expression of three tight junction proteins in the 20–50 mg/kg α‐tocopherol‐treated group was 1.5 to 4 times higher than in the control group (Figure 7 **)**.

### 3.8 The effects of α‐tocopherol on tight junction proteins of intestinal inner muscle tissues

**FIGURE 8:**
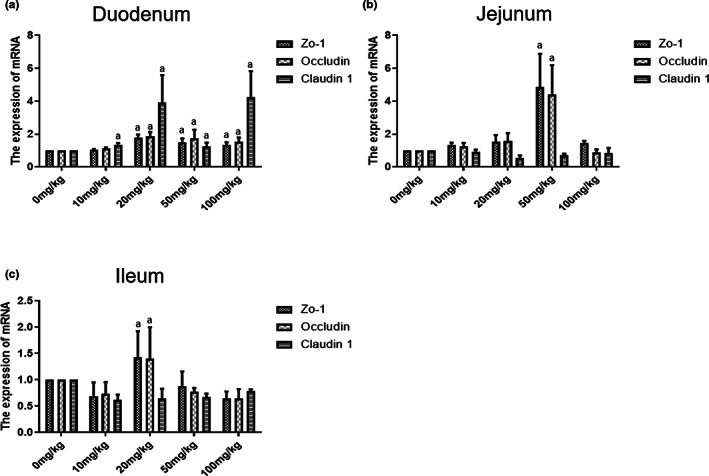
Effects of different concentrations of α‐tocopherol on tight junction proteins of intestinal inner muscle tissues. Detection of tight junction proteins such as Claudin 1, Zonula occludens 1 (Zo‐1), and Occludin expression of intestinal inner muscle tissues in weaned piglets by reverse transcription polymerase chain reaction (RT‐PCR) in duodenum (a), jejunum (b), and ileum (c). All data were represented as mean ± SD (*n* = 3, a < 0.05 vs. control).

α‐Tocopherol is having an upregulatory impact on Occludin, Claudin 1, and Zo‐1 in the duodenal inner muscle tissues at doses of 0–100 mg/kg. In the duodenum, the expression of three tight junction proteins was 1.2–3.5 times higher in the α‐tocopherol‐treated group than in the control group. In contrast, 50 mg/kg of α‐tocopherol upregulated Occludin and Zo‐1 expression in the ileum, while 20 mg/kg α‐tocopherol upregulated Occludin and Zo‐1 expression in the jejunum, indicating that α‐tocopherol is best absorbed in the duodenum (Figure 8). Thus, there is a mild reduction in the response of tight junction proteins to α‐tocopherol in intestinal inner muscle tissues compared to the mucosa, which is logical because mucosa is the first barrier that comes in contact with diet in an intact intestine. In contrast, inner muscle tissues have significantly less physical contact with diet.

## 4 DISCUSSION

The intestine is essential for body absorption and digestion. Intestinal epithelial cells act as a physiological barrier, preventing harmful antigens and microbes from entering and allowing nutrients and water to be transported from the intestinal lumen to the blood. Loss of epithelial integrity may be closely associated with tight junction dysfunction (Liu et al., 2011; Rauhavirta et al., 2014). Traditionally, α‐tocopherol has been used as an antioxidant, reducing inflammatory damage by inhibiting associated proteins or cell signaling pathways (Yang et al., 2018). Furthermore, α‐tocopherol has several pharmacological and physiological effects, including antioxidant, anticancer, and anti‐inflammatory activities (Faria et al., 2005; Tucker & Townsend, 2005). However, few studies have focused on the function of α‐tocopherol on intestinal tight junctions. Our previous work (Chen et al., 2019) demonstrated that vitamin E affects intestinal function by inhibiting cell proliferation. In the present study, we further investigate the intestinal absorption and optimal dose of α‐tocopherol. In humans, α‐tocopherol overload increases the risk of side effects. Therefore, high dose of α‐tocopherol supplements may increase all‐cause mortality. The present study found that α‐tocopherol increased the expression of tight junction proteins in the IPEC‐J2 cell line at optimal concentrations of 20–40 μmol/L. An immunofluorescence assay revealed that α‐tocopherol increases the expression of the tight junction proteins and improves the tight junction integrity.

TEER experiments confirmed that α‐tocopherol increases the epithelial cell resistance and inhibits cell permeability. These in vitro findings demonstrated that α‐tocopherol protects tight junction and tight junction proteins. α‐Tocopherol is widely used in skin lesions because of its antioxidant and anti‐inflammatory properties (Prakoeswa et al., 2018). A recent study demonstrated that α‐tocopherol reduces the triglyceride and cholesterol‐lowering effects of rice bran tocotrienol in rats fed a Western diet (Shibata et al., 2016). α‐Tocopherol promotes barrier function and anti‐inflammatory responses by binding to the regulatory domains of protein kinase Cα (PKCα), a regulator and antagonist of heart failure, and decreases the activation of the pro‐inflammatory transcription factor NF‐κB, which results in cytokines and mast cell activation (Cordero et al., 2018; Tettamanti et al., 2018). Surprisingly, our findings show that α‐tocopherol has no effect on inflammatory factors in normal small intestinal epithelial cells, implying that this increase in tight junction protein expression is irrelevant to the inflammatory pathway under normal healthy conditions. In vivo studies also demonstrated similar results. Using piglets as an animal model is advantageous because they have a higher clinical translation potential than other small animal models, and the results have a direct translational implications for the piglet feeding industry. In addition, we measured α‐tocopherol levels in serum and intestinal inflammatory factors in animal models. It was found that α‐tocopherol serum levels were not significantly higher until α‐tocopherol was added to the diet at 100 mg/kg. There was no detectable change in intestinal inflammatory factors.

Furthermore, it demonstrates that adding 0–50 mg/kg of α‐tocopherol to the diet is safe and would not cause any obvious stimulus or harm to the organs studied. The beneficial effects of α‐tocopherol on the intestinal mucosa were greater than those on inner muscle tissues, as mucosa is the first contact layer during the absorption of α‐tocopherol. Interestingly, the expression of tight junction proteins in the duodenum in the α‐tocopherol‐treated group was 2‐fold higher than in the control group, while this increase was not observed in the ileum and jejunum, indicating that the effects on the duodenum are most pronounced. The optimal dose range for the upregulatory effect of α‐tocopherol on tight junction proteins in the mucosa and inner muscle tissues is 20–50 mg/kg. We hypothesize that α‐tocopherol activates tight junction components, improving intestinal health and animal performance.

## 5 CONCLUSION

**FIGURE 9:**
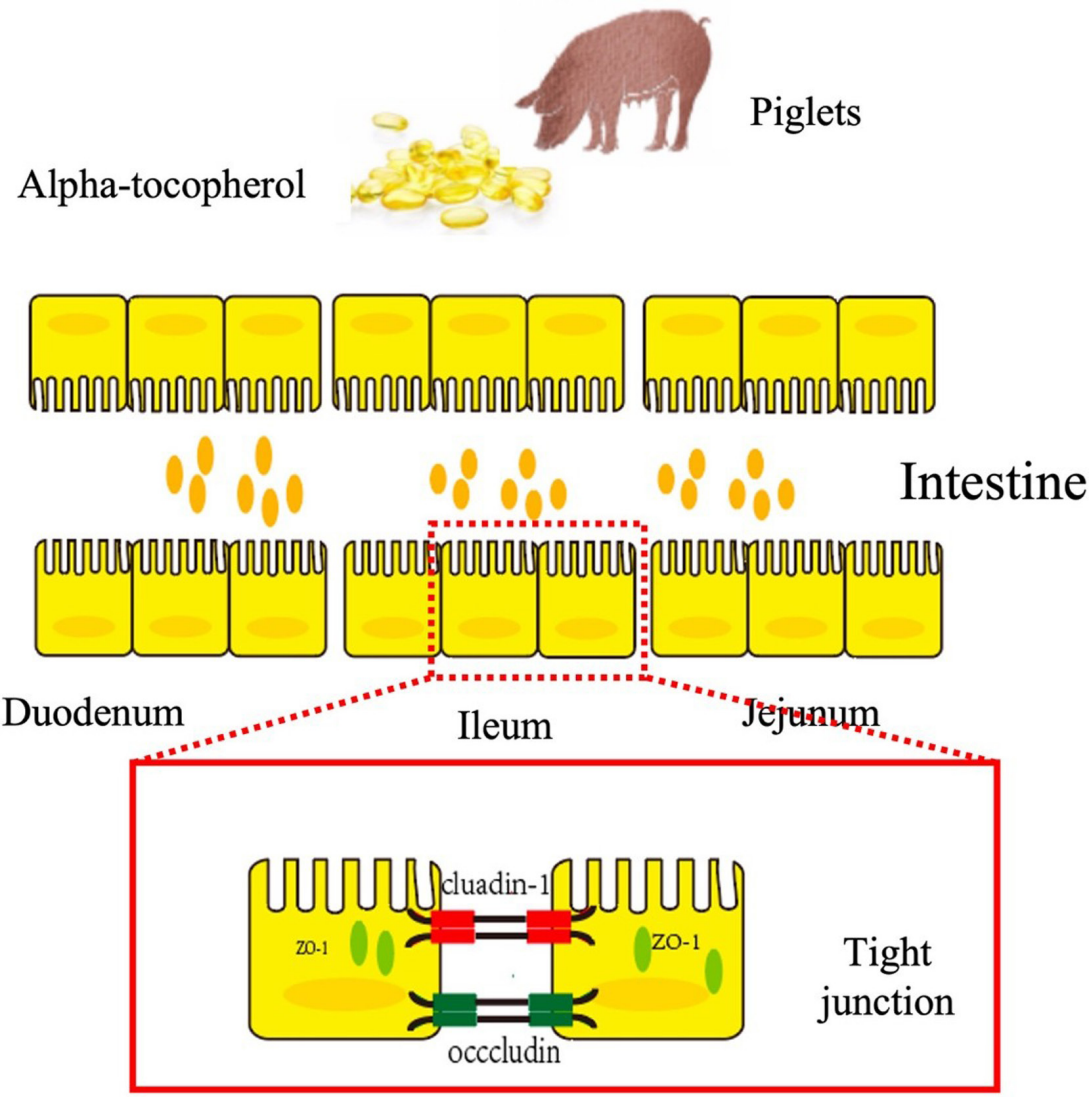
Working model describes the protective effect of α‐tocopherol on intestines in piglets. α‐Tocopherol has a profound effect on enhancing the expression of tight junction proteins in the intestinal tract and inhibiting permeability in the intestine in normal healthy conditions, both in vivo and in vitro.

In conclusion, we show that α‐tocopherol significantly enhances the expression of tight junction proteins in the intestine and inhibits permeability in the intestine under normal healthy conditions, both in vivo and in vitro (Figure 9). We can speculate that α‐tocopherol may primarily reduce oxidative stress and influence reactive oxygen species (ROS)‐related pathways independent of inflammatory signaling, particularly in normal and healthy conditions. However, the detailed mechanisms by which α‐tocopherol affects the intestinal barrier and inflammation in diseased conditions need further investigation.

## FUNDING INFORMATION

This work was supported by grants such as Institutional Open Fund (KF2022001), Key Project of Developmental Biology and Breeding from Hunan Province (2022XKQ0205) to XY and from the National Key Research and Development Program of China (2016YFD0501201) to XY and YY.
